# Eco-environmental assessment model of the mining area in Gongyi, China

**DOI:** 10.1038/s41598-021-96625-9

**Published:** 2021-09-02

**Authors:** Ying Wang, Xueling Wu, Siyuan He, Ruiqing Niu

**Affiliations:** 1 Institute of Geophysics and Geomatics, China University of Geosciences, Wuhan, 430074 China; 2 School of Environmental Science and Engineering, Southern University of Science and Technology of China, Shenzhen, 518055 China

**Keywords:** Machine learning, Ecology

## Abstract

The ecological environment directly affects human life. One of the ecological environmental issues that China is presently facing is deterioration of the ecological environment due to mining. The pollution produced by mining causes the destruction of land, water bodies, the atmosphere, and vegetation resources and new geological problems that seriously impact human civilization and life. The main purpose of this study is to present an environmental assessment model of mine pollution to evaluate the eco-environment of mining. This study added mineral species and mining types into the factor layers and built an improved evaluation system to accurately evaluate the impact of mines on the eco-environment. In the non-mining area, the grades of the eco-environment were divided according to the Technical Criterion for Ecosystem Status Evaluation standard document. In the mining area, the grades of the assessment for the eco-environment were classified by a field survey. After comparing the accuracy of various methods, the support vector machine (SVM) model, with an accuracy of 94.8%, was chosen for the mining area, and the classification and regression tree (CART) model, with an accuracy of 89.36%, was chosen for the non-mining area. Finally, environmental assessment maps for the entire study area were generated. The results indicate that the mine environmental assessment system established by this study avoids the subjective limitations of traditional assessment methods, provides an effective method for assessing ecological quality, and will help relevant departments to plan for mine resources.

## Introduction

The ecological environment refers to the sum of various natural forces (materials and energy) or actions that are closely related to human life and production activities. Mining is considered an important driving force for geological environmental problems, including geological disasters, environmental pollution, and resource destruction^1,2^. Thus, the ecological security of mining areas has become an important research topic. Mine development areas generally have complex terrains and diverse features, and obtaining information on the land features can be difficult. In recent years, researchers have used the multi-sensor, multi-spectral, and multi-temporal advantages of remote sensing (RS) technology, combined with other relevant data, to conduct dynamic surveys of the development status of mineral resources and the mining eco-environment^3^. RS technology has the characteristics of fast information acquisition, short period and large coverage, and the acquisition of information is less restricted by conditions. Therefore, it can quickly and extensively monitor the ecological environment of a mining area, which has advantages over traditional methods.

During the long-term development of mineral resources, the negative impacts of various geological environmental problems induced by mining activities on the regional eco-environment have been neglected. Thus, serious geological environmental problems have accumulated, such as landscape destruction, vegetation destruction, and water and soil loss; excessive groundwater drainage, resulting in a decline in the regional groundwater level and destruction of the water resource balance; excessive land use for waste soil, waste rock, and waste slag; and ground subsidence, ground fissures, mountain collapse, landslides, and other secondary geological disasters^4^. The more serious geological environmental problems that have occurred in mines are mainly distributed in Gongyi city, Henan Province, China, as follows: (1) open-pit mines, which have a large number of extraction wells, cause extremely serious land destruction; (2) large amounts of stripped waste are randomly discharged, and mudslides are easily triggered in the rainy season; and (3) cracks in the mountains are liable to induce collapses and landslides, in addition to other serious geo-environmental problems and hidden hazards^5^. Large amounts of tailings are used for stripping and processing gravel, and along the slope, landslides, and debris flow disasters are easily triggered; in the mining and processing areas, the environment is seriously damaged, and dust pollution is prominent.

Relying on conventional methods, such as ground surveys, is very expensive and time consuming for determining the source and extent of environmental pollution^6^. Zhao et al. studied the level of heavy metal pollution by measuring the chemical composition of soil in coal mining areas^7^. However, the chemical measurement method is not practical for large-scale detection. The continuous improvement of geographic information system (GIS) applications and RS has increased opportunities for environmental data analysis^8^. Many studies have used GIS and RS for environmental assessment, and many methods can be combined with GIS and RS for environmental evaluation^9^. Xu et al. took the coal mining area as a case area and presented a method of monitoring and assessing landscape ecological quality using a landscape ecological assessment model based on landscape ecological theories, remote sensing and geographic information systems technology^10^. Studies in Morocco by Khalil et al.^11^ have shown that integrating multi-disciplinary data and using GIS techniques is more efficient than traditional methods in assessing the impact of abandoned mines on the environment and predicting the process of pollution. For the research conducted at the Dexing mine, Jiangxi Province^12^, the water colour, mine acreage and mineral residue locations and the variation in the plant coverage within 1 km around the water system were defined as the index indicators. The methods used to calculate these index indicators are also illustrated and are based on primary geographical information and RS data. For example, digital elevation model (DEM) data are often included in the ASTER GDEM V2 dataset, and land type information can be obtained from most RS images.

The quality of the environment can be evaluated in many ways. Traditional methods, such as fuzzy comprehensive evaluation (FCE), the comprehensive index method (CIM), and the analytic hierarchy process (AHP), are widely used in multi-factor comprehensive evaluation methods because of their ease of handling^13–15^. For example, the AHP model was combined with GIS, and the coastal mining city Longkou was used as a research area to evaluate the geological and eco-environmental quality based on spatiotemporal big data^16^. However, these evaluation results are subjective in determining the evaluation weights^17^. Wang and Li used the influence of the mining construction process, natural eco-environment, and social economic environment as the indicators in the mining environment assessment, using rank correlation analysis (G1 method) to obtain the assignment for weight and build a single-mine environmental quality evaluation system^18^. The support vector machine (SVM) model and the classification and regression tree (CART) model are also often used to generate predictions based on large amounts of data. For instance, the SVM algorithm was designed to classify land use types in mining areas and provides good classification accuracy^19^. This reflects the advantages of the SVM in classification problems. In terms of environmental assessment, a study on soil environmental assessment in Guizhou, China, found that the SVM model was superior to the FCE and Nemerow comprehensive pollution index methods because it is more in line with the actual situation^20^. Li Dong et al. concluded that the SVM model was less prone to errors and faster than the back propagation (BP) neural network model when simulating the nonlinear relationship between various factors in the mine and conducting environmental evaluation^21^. The CART model can help improve the accuracy of metal contamination assessments to nearly 90% and can be used to study the relationship between metal pollution and human variables^22^. Since these machine learning methods have shown their advantages of low manual intervention and high efficiency, they have been increasingly used in environmental assessment.

In this study, the main purpose was to propose a mine pollution environment assessment model. Taking the Gongyi city mining area as an example, a widely used intelligent algorithm evaluation model was obtained. After using remote sensing images to interpret the mine information, DEM, soil type, land use type and meteorological data were used to generate the factor layer after preprocessing. Mineral types and mining types were added to the factor layer, and an improved evaluation system was established. The entire study area was divided into two parts, the mining area and the non-mine area, according to the mining area and government planning. Different intelligent models were used to predict environmental assessments across the whole study area. This method avoids the limitations of traditional sampling methods and reduces the subjectivity of empirical models. This work provides an efficient method for evaluating ecological quality and will help future resource development and mining planning.

## Study area

**Figure 1:**
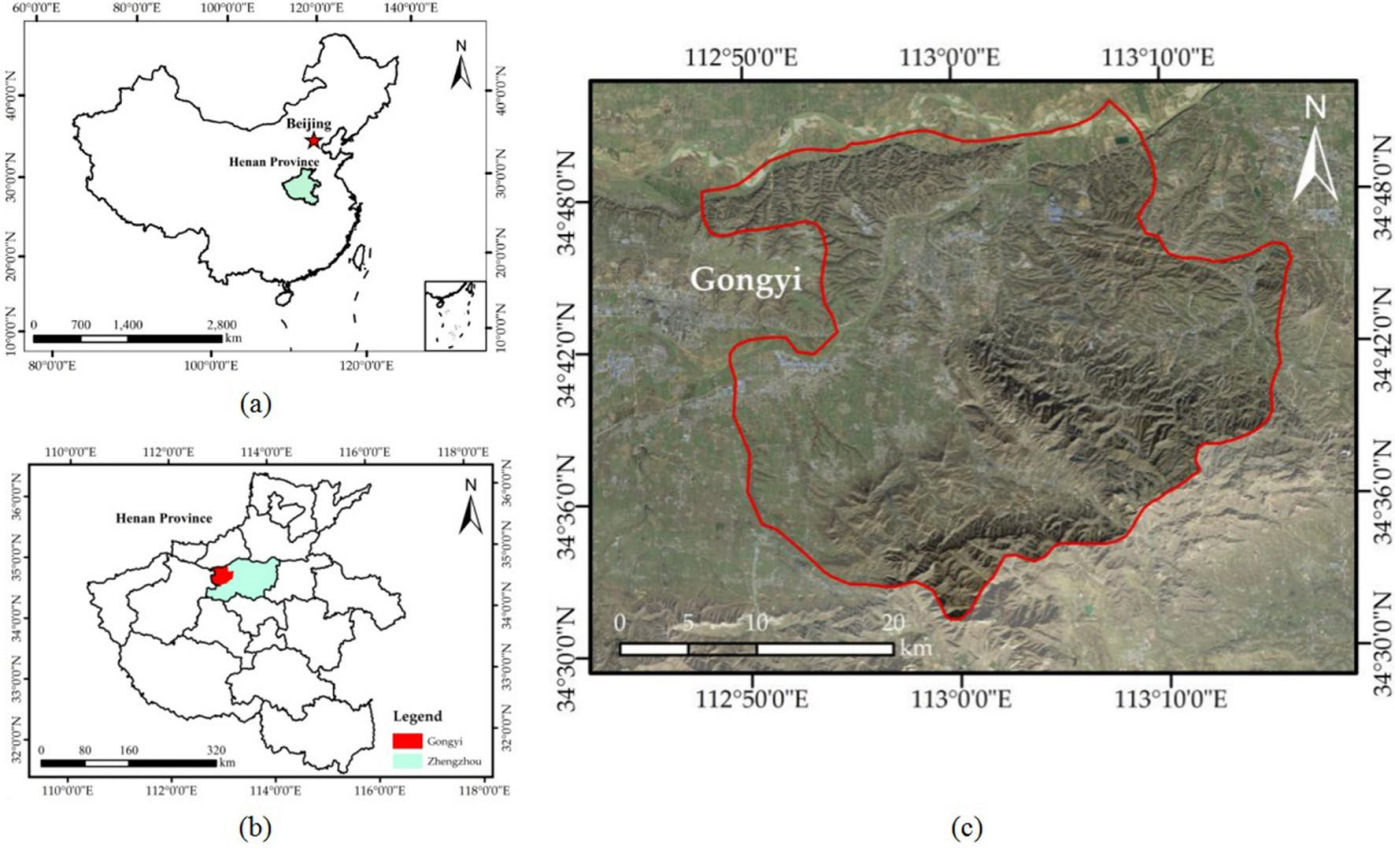
Location of the study area in China. (**a**) Map of China; (**b**) map of Henan Province; and (**c**) Gaofen-2 (GF-2) satellite image (http://www.hbeos.org.cn/) showing the location of Gongyi. The Figure is created using ArcGIS ver.10.3 (https://www.esri.com/).

Zhengzhou city is the capital of Henan Province, located in the hinterland of the Central Plains and among the nine states of China (Fig. 1b), and it is a political, economic, and cultural centre in Henan Province. Zhengzhou is known as the “heart” of the railways of China, and its road traffic extends in all directions. Additionally, Zhengzhou city is rich in mineral resources^23^; mining accounts for an important proportion of the total industrial output from the city and has become the main industrial economic pillar of Zhengzhou city. Gongyi city is part of Zhengzhou city and is located in the northern foothills of Mount Songshan, 82 kms east of Zhengzhou (Fig. 1c). Gongyi is situated between latitudes 34°31′–34°52′N and longitudes 112°49′–113°17′E and is 43 kms long from east to the west and 39.5 kms wide from north to south, with a total area of 1052 km^2^^24^. Gongyi city is located in a warm temperate continental monsoon climate. The annual sunshine time is approximately 2400 h, and the average temperature is between 14.3 and 14.8 °C, which can meet the industrial production demand^25^. The average annual precipitation is 583 mm, and the precipitation is mostly concentrated in July, August and September (accounting for 70% of the whole year). In terms of landform types, Gongyi is low in the north and high in the south, with mountains, hills, and plains. Gongyi city is rich in mineral resources. At the end of 2017, 21 types of minerals were discovered in the city, including coal, iron ore, bauxite, gallium ore, limestone, refractory clay, ceramic clay, pyrite, bauxite, etc. The main problems of the mine geological environment in Gongyi are the destruction of the topography and landscape caused by open and underground mining, the occupation and destruction of land resources, the geological disasters induced by mining activities, and the destruction of aquifers by underground mining activities. The four maps in Fig. 1 show the geographical locations of China, Henan Province, Zhengzhou and Gongyi (Fig. 1).

## Data

### Mine data

**Figure 2:**
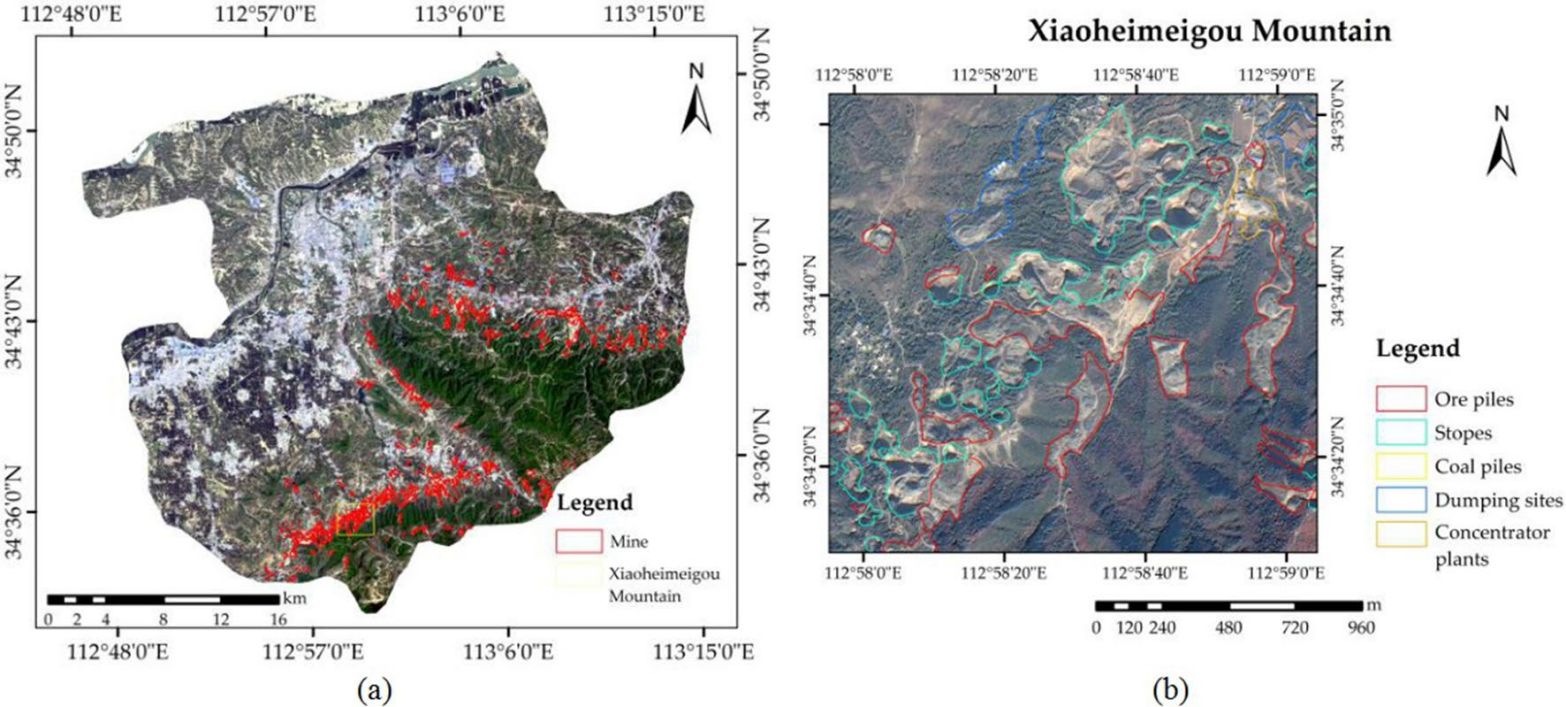
Mine interpretation and field investigation. (**a**) Map of Gongyi; (**b**) maps of the mine interpretation on Xiaoheimeigou Mountain. The Figure is created using ArcGIS ver.10.3 (https://www.esri.com/).

Mine data, such as mine distribution data, mine type data, and mining method data, were obtained by visual interpretation from Gongyi’s high spatial resolution RS image data. High-resolution remote sensing data show the true shape, size, colour and other information of objects on the surface of the earth. The texture information of ground objects is abundant and can be used to better analyse dynamic changes and monitor the environment. These RS image data were collected from the GF-2 satellite, which is the first civil optical RS satellite in China, with a spatial resolution up to 0.8 m, independently developed by China. The resolution of GF-2 data is 0.81 m for panchromatic data and 3.24 m for multi-spectral data^26^. This study used a high-quality GF-2 RS image on May 21, 2016, because the image of this day has no cloud coverage and high image quality in this area. The impact of mining on surface changes is not obvious on satellite images. The information on the scale and type of mining reflected by a high-quality remote sensing image is representative for a long period of time, so we only used a clear image to acquire the mining information. This RS image was then preprocessed using atmospheric correction, orthorectification, and geometric correction. After the fusion process, the final image spatial resolution is 1 m. The government provided the mining scope, minerals (metallic minerals or non-metallic minerals) and mining methods (open-pit mining or underground mining) reported by mine owners. The metal mines in Gongyi city are mainly bauxite and iron ore, and the non-metallic mines are mainly coal mines, with a few limestone mines. We conducted the visual interpretation of the mine on the GF-2 RS images according to the minerals and mining methods within the specified mining scope. Visual interpretation relies on the knowledge and experience of the interpreter to identify the mine type and range information from the remote sensing images, establish the interpretation marks, use ArcGIS software to draw out the mining area vector and set the attribute table. In the GF-2 RS image, the spots of different mine types differ in colour, size, and texture, and some have obvious mining equipment and houses. Then, 100 training sampling points were randomly selected. To ensure that each mine type was included, we randomly sampled each type proportionally. After the field investigation at the sampling points, the interpretation marks of each type of mine were summarized, and all original interpretation results were modified. In the modified interpretation map, we obtained the following results: the mine types in the metal mine mainly include stopes, concentrator plants, ore piles, and tailings ponds, and the mine types in the non-metallic mine mainly include stopes (open pits), concentrator plants, dumping sites, coal piles, and ore piles (Fig. 2).

### Socio-natural factors

**Figure 3:**
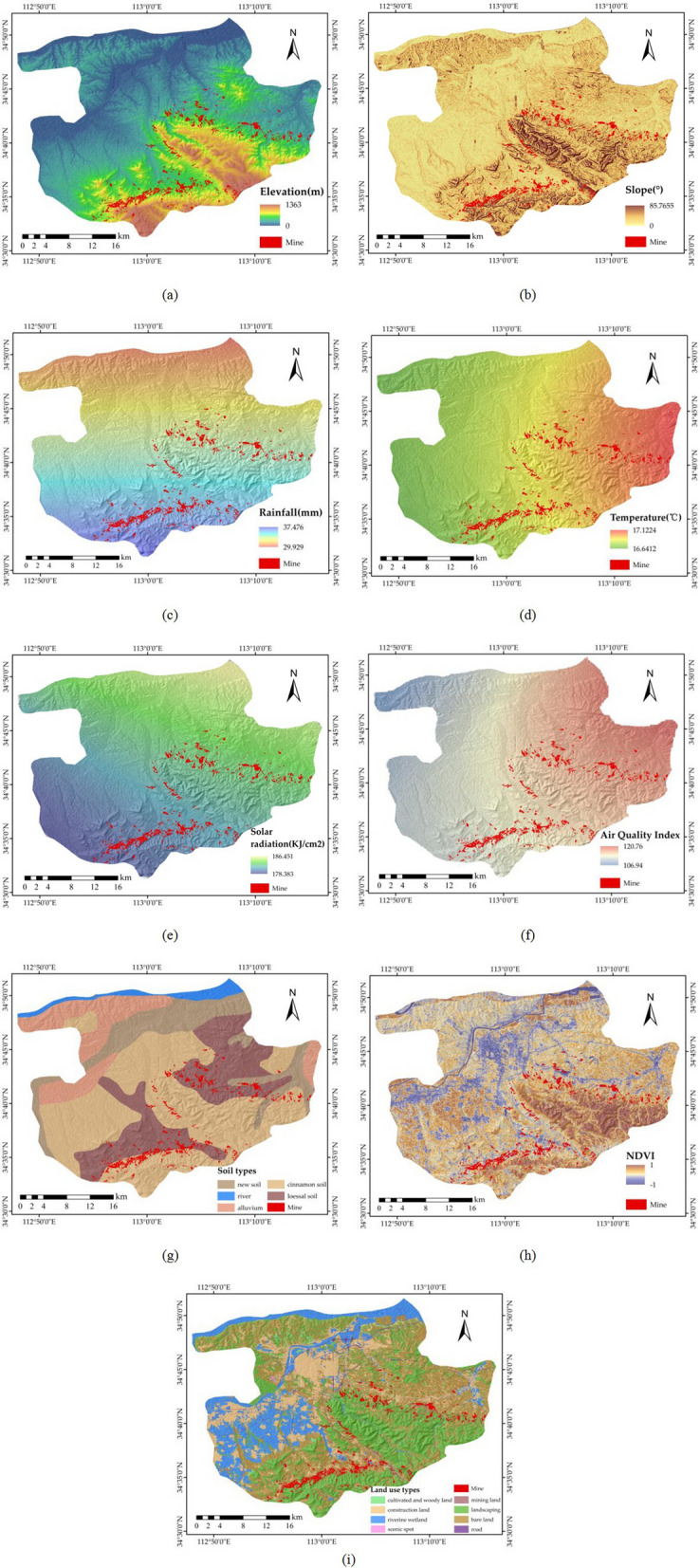
Socio-natural factors. (**a**) Elevation; (**b**) slope; (**c**) rainfall; (**d**) temperature; (**e**) solar radiation; (**f**) air quality index; (**g**) soil type; (**h**) NDVI; and (**i**) land use types. The Figure is created using ArcGIS ver.10.3 (https://www.esri.com/).

Social and natural factors were gathered according to the official government document HJ 192–2015 on the eco-environment assessment. These factors included DEM-derived factors, meteorological-derived factors, and land type factors (Fig. 3). The DEM data were obtained by processing the ASTER GDEM V2 dataset and have a resolution of 30 m. Elevation and slope data were extracted from the DEM data by using the ENVI and ArcGIS software packages. Meteorological-derived factors included rainfall, temperature, solar radiation, and air quality index (AQI) data^27^. These meteorological data were collected from March 2016 to February 2017. Then, the kriging method in ArcGIS software was used to interpolate the meteorological data of the site. The kriging method was used because it is a multi-step process that includes exploratory statistical analysis of the data, modelling of variograms, and creation of surfaces, as well as the study of variance surfaces. Thus, kriging is an accurate and adaptable method^28^. Through simple comparison, we find that the results obtained by setting the 12 interpolation points using the spherical model of the kriging method are more consistent with the general meteorological state of the study area. Figure 3 shows the first season (from March 2016 to May 2016) as an example. The land type factors included soil type, normalized difference vegetation index (NDVI) and land use type. The soil types and land use types were obtained, and the soil type map scale was 1:200,000^29^. The soil types shown in Gongyi include new soil, fluvo-aquic soil, cinnamon soil, and loess soil according to the “Classification and codes for Chinese soil GB/T 17296-2009”, a government standard document published in 2009. NDVI was abstracted from Landsat 8 OLI_TIRS RS images on 27 July 2017 with a resolution of 30 m. The pixel size of all the factor layers was set to 30 m to ensure the feasibility of the calculation.

## Methods

**Figure 4:**
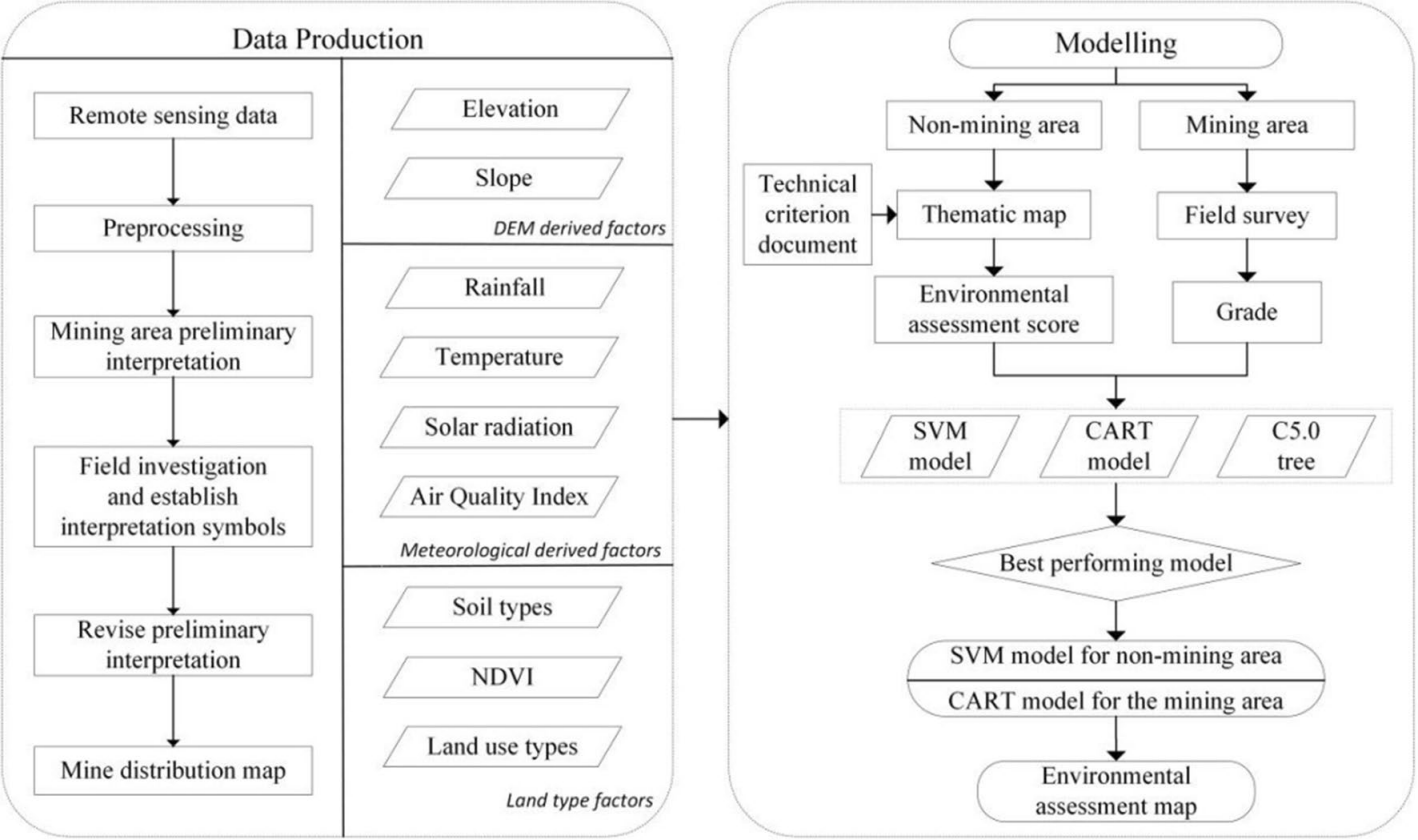
Methodological flowchart of the research process.

After collecting all the factors, establishing an eco-environmental evaluation index system has become a problem. To strengthen the protection of the eco-environment and evaluate the eco-environment and trends, the Ministry of Environmental Protection of China issued the “Technical Criterion for Ecosystem Status Evaluation”, an official document of environmental assessment^30^. This document marked and ranked the environment from 0 to 100 based on environmental quality. However, this document is applicable to only common ecological areas^31^. For mining areas and areas affected by mines, this evaluation method is not practical because this document did not take into account damage to the environment caused by mines, such as atmospheric destruction by dust formed in open pits, excessive land use for ore piles and dumping sites, and soil pollution by coal piles, mining sites, and concentrator plants^32,33^. Additionally, the probability of geological disasters can increase due to the mining surface^34^. The main purpose of this research is to propose a mining pollution environment assessment model that combines traditional experience methods and machine learning methods. On the basis of traditional methods, the intelligence, applicability and efficiency advantages of machine learning algorithms are brought into play. Taking the Gongyi city mining area as an example, a widely used intelligent algorithm evaluation model was obtained. After using the remote sensing image to interpret the mine information, DEM, soil type, land use type and meteorological data were used to generate the factor layer after preprocessing. The whole study area was divided into mining and non-mining areas according to the extracted mining zones and distance range affected by the mining area. This range was obtained from a buffer zone of 1000 m around the centre of the developed mining area and key eco-environment protection area. The government defines the eco-environment protection area as a visual range of important traffic trunks, rivers, and lakes. In the non-mining area, the environmental assessment scores were obtained according to the official document, which provides the calculation formula and score weight of the indicator layers. In the mining area, developing a formula with good applicability to all ranges is difficult. Therefore, intelligent algorithms were used to obtain the score of the whole region. Intelligent algorithms have unique advantages in solving the problem of optimal solutions. After a test, an additional comparison was made among the SVM model, CART model, and C5.0 tree model (Fig. 4).

### SVM model

An SVM is a new general learning method based on statistical learning theory. This method can effectively perform accurate fitting of high-dimensional nonlinear systems based on small samples and adopts the principle of minimum structural risk, which has good generalization^35^. An SVM is one of the most commonly used and best-performing classifiers because of its excellent generalization ability. The SVM optimization goal is to minimize the risk of the structure, rather than the least risk, thus reducing the data size and data distribution requirements, which is helpful in the field of environmental assessment. However, an SVM is not better than any other algorithm in any scenario because it is difficult and time consuming for an SVM to train large amounts of data, so there are some limitations for a wide range of real-time evaluation analyses. At present, SVMs have been well applied in the fields of time series analysis, regression analysis, cluster analysis, signal processing, image classification, and control systems, but the application of SVMs in environmental assessment has just started.

Training data sets, i.e., D = {(x_1_,y_1_)…,(x_l_,y_l_) }, where l is the sample size and D is the training set, are used to estimate the regression function in the linear function set^36^:

1 $${{f}}\left( {{{x}},{{a}}} \right) \, = \omega {{x }} + {{ b}}$$

The SVM algorithm minimizes function (2) under constraint (3) using the principle of structural risk minimization.

2 $$\Phi \left( {\omega ,\xi_{i}^{ + } ,\xi_{i}^{ - } } \right) = \frac{1}{2}\left( {\omega \cdot \omega } \right) + c\sum\limits_{i = 1}^{l} {\left( {\xi_{i}^{ + } ,\xi_{i}^{ - } } \right)}$$

3 $$\left\{ \begin{aligned} & y_{i} - \left( {\omega \cdot x_{i} } \right) - b \le \xi_{i}^{ + } + \varepsilon \hfill \\ & \left( {\omega \cdot x_{i} } \right) + b - y_{i} \le \xi_{i}^{ - } + \varepsilon \hfill \\ & \xi_{i}^{ + } \ge 0 \hfill \\ & \xi_{i}^{ - } \ge 0 \hfill \\ \end{aligned} \right.\;\;i \, = \, 1,2,...,l$$

In this formula, c is the penalty coefficient, which determines a compromise between empirical risk error and model complexity; ξ_i_^+^ and ξ_i_^−^ are relaxation factors; ε is the allowable error; b is the offset; l is the number of training samples; and ω is the weight vector. By constructing the Lagrange function, the dual problem of the original function is obtained, and the above problem eventually becomes a convex quadratic programming problem.

### CART model

The CART model is a nonparametric nonlinear regression method proposed by Breiman et al.^37^. The basic principle of the CART model is a binary decision tree structure formed by cyclic analysis of training data sets composed of test variables and target variables. The CART algorithm uses the Gini index as the criterion for selecting the best test variable. The Gini index is defined as follows:

4 $$Gini \; Index = 1 - \sum\limits_{j}^{J} {p^{2} \left( {j/h} \right)}$$

5 $$P\left( {j/h} \right) = \frac{{n_{j} \left( h \right)}}{n\left( h \right)},\sum\limits_{j}^{J} {P\left( {j/h} \right) = 1}$$

In this formula, a sample from the training sample set is randomly extracted, and when a test variable has a value of h, the probability of belonging to the jth class is p(j/h); n_j_(h) is the number of samples belonging to the jth class when the test variable value is h in the training sample; n(h) is the number of samples in the training sample whose test variable value is h; and j is the number of categories.

Learning samples are required to build and evaluate the CART decision tree before using the CART model for prediction. The CART model uses the learning sample set of the following structure:

6 $$L: = \left\{ {X_{1} ,X_{2} , \ldots ,X_{m} ,Y} \right\}$$

7 $$X_{1} : = \left( {x_{11} ,x_{12} , \ldots ,x_{{1t_{1} }} } \right), \ldots ,X_{m} : = \left( {x_{m1} ,x_{m2} , \ldots ,x_{{mt_{n} }} } \right)$$

8 $$Y: = \left( {y_{1} ,y_{2} , \ldots ,y_{k} } \right)$$

X_1_–X_m_ are attribute vectors, and their attributes are continuous or discrete. Y is the label vector, and its attributes can also be continuous or discrete. When Y is a continuous quantity value, it is a regression tree; when Y is a discrete value, it is a classification tree.

The CART algorithm selects one attribute or a combination of multiple attributes from a plurality of input attributes of the model as a splitting variable of the tree node and divides the test variable into branches; this process is repeated to establish a sufficiently large classification tree. Then, the pruning algorithm is used to prune the tree, and a series of nested classification trees are obtained. Finally, the series of classification trees are tested with test data, and the optimal classification tree is selected. The CART algorithm can be used for classification and regression, and it has considerable robustness and scalability in dealing with abnormal data. For example, when certain monitoring data are missing or abnormal, this algorithm will not allow the outliers to damage the final result. However, it is difficult for this algorithm to make predictions with continuous data^38^.

The C5.0 algorithm is also an algorithm in the decision tree model family and has certain similarities with the CART algorithm. The growth process of the C5.0 decision tree uses the principle of the maximum information gain rate for node selection and splitting point selection. The C5.0 model is very robust for missing data and problematic fields and is easier to understand and explain than some other types of models^39^. However, the calculation process of this algorithm is too complicated when calculating the information gain rate, and it is prone to overfitting and decision tree bias.

## Results

### Technical criterion for ecosystem status evaluation

On March 3, 2015, the Ministry of Ecology and Environment of the People’s Republic of China approved the "Technical Criterion for Ecosystem Status Evaluation" as the national environmental protection standard. This standard is based on the former standards released in 2006, and 48 relevant documents from 2006 to 2012 were searched to propose new standards and factor weights based on actual utilization effects and expert guidance. The eco-environment assessment uses a comprehensive index (eco-environmental status index, EI) to reflect the overall state of the regional eco-environment. The indicator system includes the biological abundance index, vegetation coverage index, river density index, land stress index, and pollution loading index. These indexes reflect the abundance of organisms in the evaluated area, the level of vegetation coverage, the abundance of water, the intensity of land stress, and the extent of the pollution load. Each indicator was calculated according to its weight to obtain an eco-environment assessment map (Table 1). All parameters involved in the calculation are derived from this standard.

**Table 1:** Weights of the evaluation indicators.

Index	Biological abundance index	Vegetation coverage index	River density index	Land stress index	Pollution loading index
Weight (%)	0.35	0.25	0.15	0.15	0.10

The calculation of the eco-environment status is as follows:

9 $$\begin{aligned} EI & = 0.35*biological \; abundance \; index + 0.25*vegetation \; coverage \; index \hfill \\ & \quad + 0.15*river \; density \; index + 0.15*(1 - land \; stress \; index) \hfill \\ & \quad + 0.1*(1 - pollution \; loading \; index) \hfill \\ \end{aligned}$$

#### Biological abundance index

The biological abundance index refers to the number of certain organisms in this area. The calculation method is as follows:

10 $$Biological \, abundance \, index \, = \, \left( {BI \, + \, HQ} \right)/2$$

In this formula, BI is the biodiversity index and HQ is the habitat quality index. When the biodiversity index does not have dynamic data updates, the change in the biological abundance index is equal to the change in the HQ.

Biodiversity is a general term for the complexity of species and their genetic variation and ecosystems in space over time. Biodiversity plays an important role in maintaining soil fertility, ensuring water quality, regulating the climate, stabilizing the environment, and maintaining ecological balance.

The BI method is as follows:

11 $$BI = NPP_{mean} *F_{pre} *F_{tem} *(1 - F_{alt} )$$

NPP_mean_ is the net primary productivity. F_pre_ is the annual average precipitation. F_tem_ is the temperature parameter. F_alt_ is the altitude parameter.

NPP refers to the amount of organic matter accumulated per unit area and unit time of green plants. NPP is the remainder of the total amount of organic matter produced by photosynthesis after deducting autotrophic respiration and is usually expressed as dry weight. In this study, the estimation of NPP was based on the absorbed photosynthetically active radiation (APAR) and actual light-use efficiency (LUE) (ε) of the CASA ecosystem model^40^. The CASA model is a process-based remote sensing model that couples ecosystem productivity and soil carbon and nitrogen fluxes, driven by gridded global climate, radiation, soil, and remote sensing vegetation index datasets^41^. The model can be expressed generally as follows:

12 $$NPP(x,t) = APAR(x,t)*\varepsilon (x,t)$$

The entire study area is divided into 11,303 pixels on a 30 * 30 m grid. x indicates the location of each pixel, and t indicates time; the data were collected once a month. APAR(x,t) represents the photosynthetically active radiation absorbed by pixel x in that month (gC * m^−2^* month^−1^). Ɛ(x, t) is LUE (gC * MJ^−1^) of the vegetation^42^.

Estimation of the fraction of APAR using RS data is based on the reflection characteristics of the vegetation on the infrared and near-infrared bands. The value of APAR is determined by the effective radiation of the sun and the absorption ratio of the vegetation to the effective photosynthetic radiation. The formula is as follows:

13 $$APAR(x,t) = SOL(x,t)*FPAR(x,t)*0.5$$

where SOL(x,t) represents the total amount of solar radiation at pixel x in month t, FPAR(x,t) represents the absorption ratio of the vegetation layer to the incident photosynthetically active radiation, and a constant of 0.5 indicates the ratio of the effective solar radiation that can be utilized by the vegetation to the total solar radiation.

Since there is a linear relationship between FPAR and NDVI within a certain range, this relationship can be determined according to the maximum and minimum values of a certain vegetation type NDVI and the corresponding FPAR maximum and minimum values.

14 $$FPAR(x,t) = \frac{{(NDVI(x,t) - NDVI_{i,\min } )}}{{(NDVI_{i,\max } - NDVI_{i,\min } )}}*(FPAR_{\max } - FPAR_{\min } ) + FPAR_{\min }$$

where NDVI_max_ and NDVI_min_ correspond to the NDVI maximum and minimum values of the ith planting type, respectively. There is also a good linear relationship between FPAR and the simple ratio index (SR) of vegetation, which is represented by the following formula:

15 $$FPAR(x,t) = \frac{{(SR(x,t) - SR_{i,\min } )}}{{(SR_{i,\max } - SR_{i,\min } )}}*(FPAR_{\max } - FPAR_{\min } ) + FPAR_{\min }$$

where the values of FPAR_min_ and FPAR_max_ are independent of vegetation type and are 0.001 and 0.95, respectively; SR_i,max_ and SR_i,min_ correspond to the 95% and 5% percentiles, respectively, of the ith NDVI. SR(x,t) is represented by the following formula:

16 $$SR(x,t) = \frac{1 + NDVI(x,t)}{{1 - NDVI(x,t)}}$$

A comparison of the estimated results of FPAR-NDVI and FPAR-SR shows that the FPAR estimated by NDVI is higher than the measured value, while the FPAR estimated by SR is lower than the measured value, but the error is less than that estimated directly by NDVI. As a result, these two values can be combined, and their weighted average value is taken as an estimate of the estimated FPAR, while ɑ means weight:

17 $$FPAR(x,t) = \alpha FPAR_{NDVI} + (1 - \alpha )FPAR_{SR}$$

**Figure 5:**
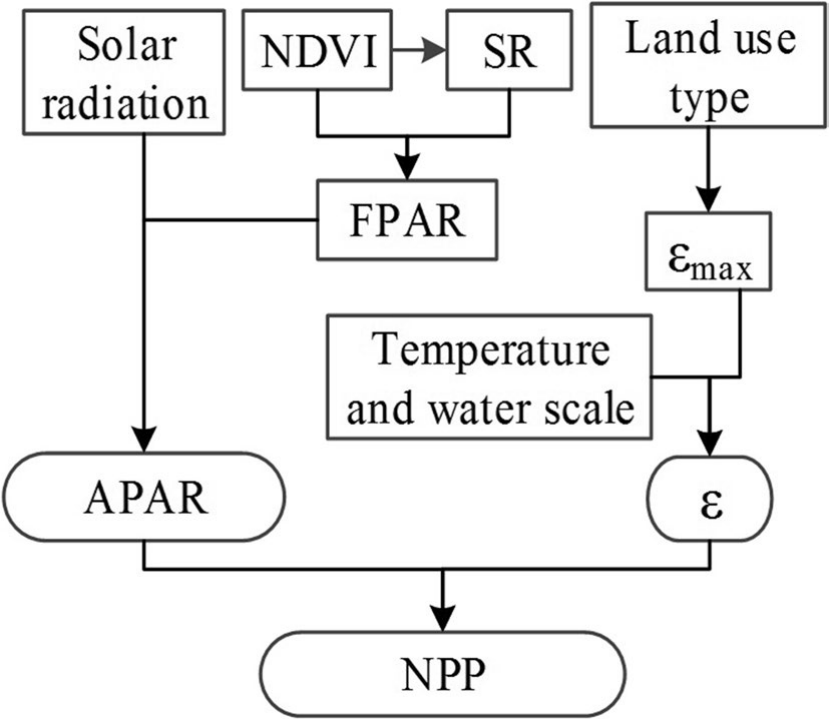
NPP calculation process.

**Figure 6:**
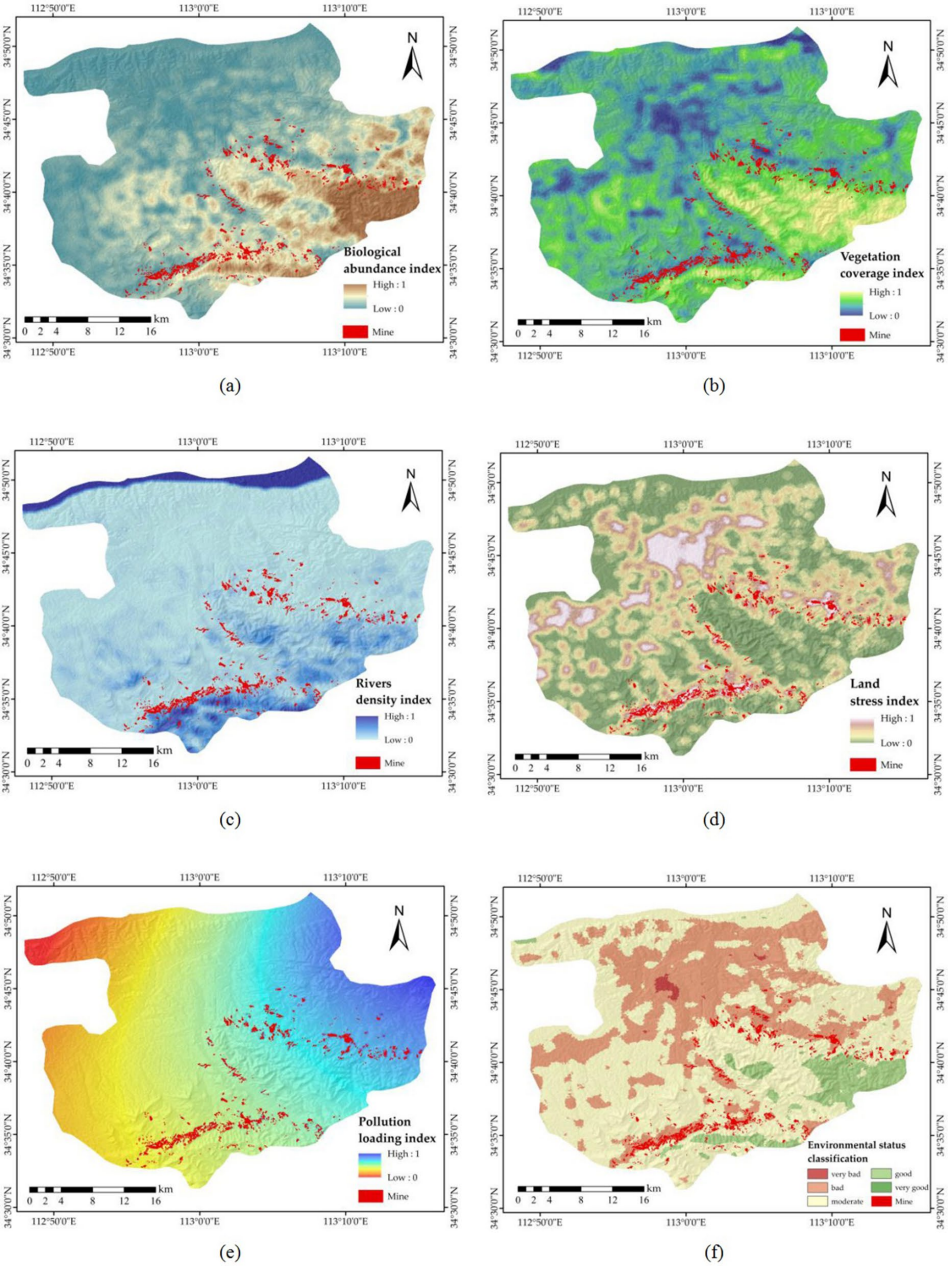
Eco-environment assessment indexes and evaluation rating map (the first quarter was used as an example). (**a**) Biological abundance index; (**b**) vegetation coverage index; (**c**) river density index; (**d**) land stress index; (**e**) pollution loading index; and (**f**) environmental status classification. The Figure is created using ArcGIS ver.10.3 (https://www.esri.com/).

Light use efficiency (LUE) refers to the ratio of chemical energy contained in organic dry matter produced per unit area over a certain period of time to the photosynthetically active radiation absorbed by plants projected onto the same area at the same time. Different vegetation types and the same types of vegetation have different light energy utilization rates in different living environments^43^. The differences are mainly due to the characteristics of the vegetation itself, temperature, moisture, and soil^44^. Vegetation has the highest utilization rate of light energy under ideal conditions, but the maximum light energy utilization rate in the real environment is mainly affected by temperature and moisture, which can be expressed as follows:

18 $$\varepsilon \left( {x,t} \right) = T_{\varepsilon 1} \left( {x,t} \right) \cdot T_{\varepsilon 2} \left( {x,t} \right) \cdot W_{\varepsilon } \left( {x,t} \right) \cdot \varepsilon_{\max }$$

where T_ε1_(x,t) and T_ε2_(x,t) represent the stress effects of low temperature and high temperature on light energy utilization, respectively, W_ε_(x,t) is the effect of water stress on the maximum light energy utilization under ideal conditions, and ε_max_ is the maximum light energy utilization under ideal conditions (gC * MJ^−1^). The maximum solar energy utilization rate ε_max_ varies depending on the vegetation type. In this study, the maximum light energy utilization rate of different land use types simulated by an improved Carnegie-Ames-Stanford Approach (CASA) model is used as the input parameter of light energy utilization in the CASA model (Table 2). The monthly maximum light energy utilization rate is determined in three steps: first, calculate the APAR, temperature, and water stress factors of all pixels; then, select the NPP measured data of the same time period in the study area; finally, simulate the ε_max_ of vegetation according to the principle of minimum error^45^. Figure 5 shows the calculation process of NPP. The weight of each habitat type in the HQ is shown in Table 3. The weight value is derived from the official document^30^. To facilitate the calculation, this paper normalizes the calculation results from 0 to 1 (Fig. 6a).

**Table 2:** Maximum LUE rates of different land use types.

Land use types	Woodland	Shrub	Sparse woodland	Grassland	Water	Construction land	Arable land
ε_max_	0.638	0.429	0.475	0.542	0.542	0.542	0.542

**Table 3:** Weight of each habitat type in the HQ.

Land use types	Weights (%)	Habitat type	Weights (%)
Woodland	0.35	Woodland	0.60
		Shrubbery	0.25
		Sparse woodland and other woodland	0.15
Grassland	0.21	High coverage grass	0.60
		Medium coverage grass	0.30
		Low coverage grass	0.10
Water wetland	0.28	River	0.10
		Lake	0.30
		Beach wetland	0.50
		Permanent glacier snow	0.10
Arable land	0.11	Paddy field	0.60
		Rainfed cropland	0.40
Construction land	0.04	Urban construction land	0.30
		Rural settlement	0.40
		Other construction land	0.30
Unutilized land	0.01	Sandy land	0.20
		Saline-alkali land	0.30
		Bare land	0.20
		Bare rock gravel	0.20
		Other unused land	0.10

#### Vegetation coverage index

The vegetation coverage index was obtained from the NDVI, which is a simple, effective, and empirical measure of surface vegetation status. The vegetation index mainly describes the difference between the reflection of vegetation in the visible and near-infrared bands and the soil background. This index also reduces the solar elevation angle and noise caused by the atmosphere and is thus the most widely used and effective calculation method. Each vegetation index can be used to quantitatively describe the growth of vegetation under certain conditions. The expression is as follows:

19 $$NDVI = \frac{NIR - R}{{NIR + R}}$$

where NIR and R are reflectance values in the near-infrared and red bands, respectively.

NDVI values are obtained by processing the RS images of the Landsat 8 satellite. This satellite is equipped with an operational land imager (OLI) that includes nine bands with a spatial resolution of 30 m, including a 15-m panchromatic band. To facilitate the calculation, this paper normalizes the calculation results from 0 to 1 (Fig. 6b).

#### River density index

The river density index refers to the total length of rivers, lakes, and water resources in the assessed area as a percentage of the assessed area, which is used to reflect the abundance of water in the assessed area and is calculated as follows:

20 $$\begin{gathered} River \; density \; index = (A_{riv} *river \; length / area + A_{lak} *water \; area/area \hfill \\ + A_{res} {*}amount \; of \; resources/area \, )/3 \hfill \\ \end{gathered}$$

where A_riv_ is the normalization coefficient of river length, with a reference value of 84.3704, A_lak_ is the normalization coefficient of the lake area, with a reference value of 591.7909, and Ares is the normalization coefficient of water resources, with a reference value of 86.387. Finally, the calculation results were normalized from 0 to 1 (Fig. 6c).

#### Land stress index

The land stress index is the degree to which the land quality in the assessment area is under stress. The weight of the land stress index evaluation is shown in Table 4.

**Table 4:** Weight of the land stress index evaluation.

Type	Severe erosion	Moderate erosion	Construction land	Other land stress
Weight (%)	0.4	0.2	0.2	0.2

The calculation method is as follows:

21 $$\begin{gathered} Land \; stress \; index = A_{ero} *(0.4*severe \; erosion \; area + 0.2*{\text{mod}}erate \; erosion \; area \hfill \\ + 0.2*construction \; land \; area + 0.2*other \; land \; stress \; area)/area \hfill \\ \end{gathered}$$

where A_ero_ is the normalization coefficient of the land stress index, with a reference value of 236.0436. According to the “Classification criteria for soil erosion”^46^, the influencing factors of soil erosion, vegetation, soil texture, landform, and precipitation are ranked according to importance. In the calculation of the land stress index, all the land is divided into three categories, in which the weight of severe erosion is 0.4, the weight of non-erosion is 0, and the other erosion types such as moderate erosion and construction land are 0.2. The areas with severe erosion include vegetation coverage less than 30% and areas of soil erosion greater than 3.7 mm/a due to human activities. These areas are generally developed on highly erosive-sensitive soils. Cinnamon soil and loess soil in the study area are highly erosive-sensitive soils. Therefore, the industrial and mining areas of cinnamon and loess soil types are regarded as severe erosion areas. Areas with vegetation coverage greater than 50% are non-eroded areas, so water bodies and woodlands are divided into non-erodible areas. All areas except these two types have a weight of 0.2. Finally, the calculation results were normalized from 0 to 1 (Fig. 6d).

#### Pollution loading index

The pollution loading index refers to the load of pollutants in a certain area or an environmental element. In this study, the AQI was used to calculate the pollution loading index, and the results were normalized from 0 to 1 (Fig. 6e).

The eco-environmental evaluation score was calculated based on the national environmental protection standard according to the weight of each indicator (Fig. 6f).

### Improved evaluation system and intelligent evaluation model

#### Improved evaluation system

Considering that the evaluation factors in the national environmental protection standards are applicable to ordinary areas, areas affected by mines should have more evaluation factors than those in the standards. Thus, an improved evaluation system was proposed. The improved evaluation system has added factors that affect the environment of the mine based on the factors of the original system. The impact of the mine on the environment is reflected in the pollution of the atmosphere, such as dust from open pits and industrial waste from concentrators; the occupation of land by solid waste, such as ore piles and coal piles; soil pollution, such as the diffusion of heavy metals from coal piles, coal mine concentrator plants, and mines; and the increased likelihood of geological disasters, such as collapse caused by underground mining, spontaneous coal combustion and landslides caused by open-pit mining surfaces. Therefore, the improved evaluation system adds an air pollution range, a solid waste area, a geologic hazard range, and a metallic and non-metallic mine soil pollution buffer to the national environmental protection standards.

**Figure 7:**
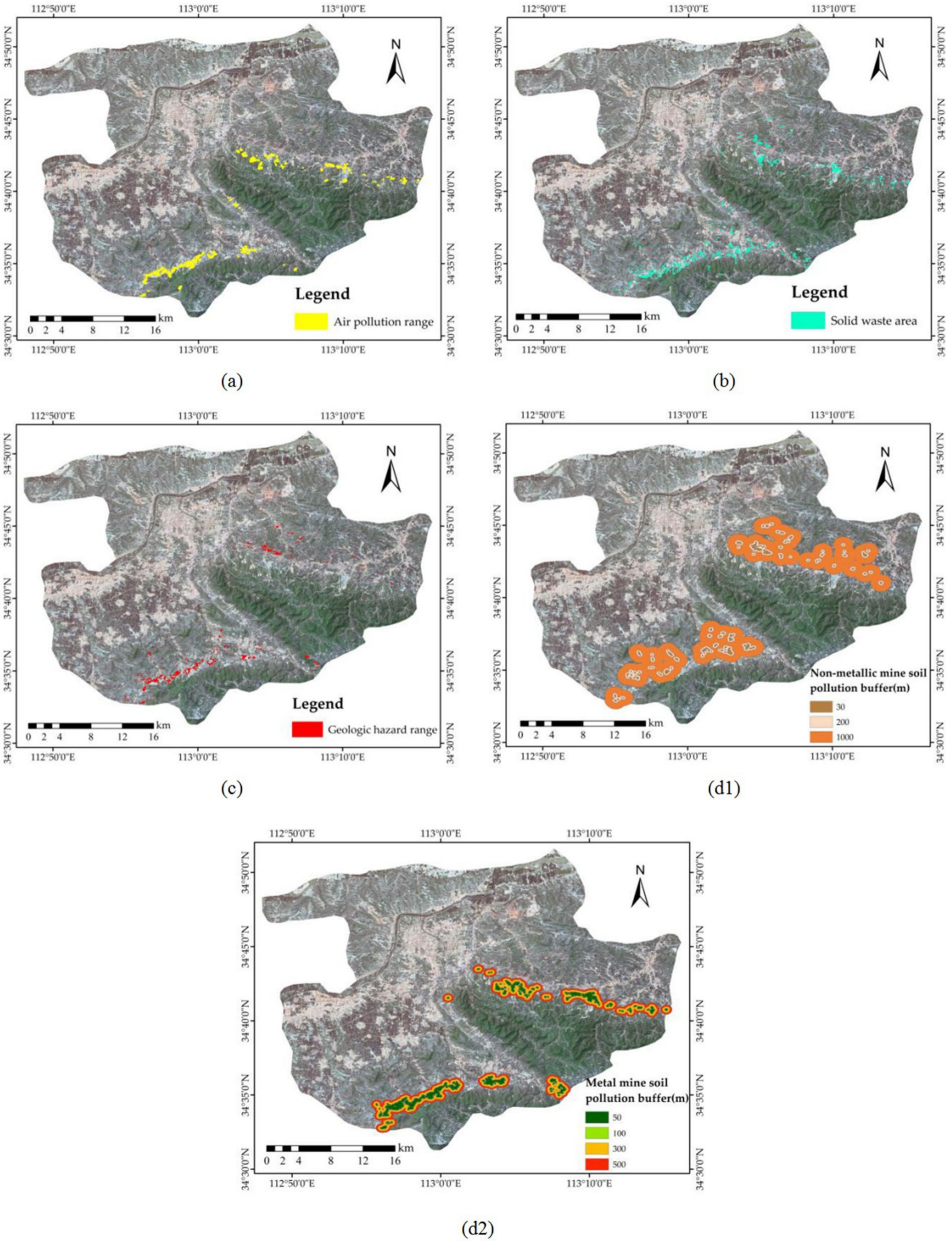
New factors in the improved evaluation system. (**a**) Air pollution range; (**b**) solid waste area; (**c**) geological hazard range; (**d1**) non-metallic mine soil pollution buffer; and (**d2**) metal mine soil pollution buffer. The Figure is created using ArcGIS ver.10.3 (https://www.esri.com/).

The area of air pollution in mining areas is generally near open pits and concentrator plants. Therefore, the air pollution range was selected within 50 m around the open pits and the concentrator plants. Due to the dilution and dispersion of the air itself, an estimate of the pollution is the reciprocal of the wind speed (Fig. 7a).

Mine solid waste pollution includes a large amount of waste rock from open-pit mining and pit mining, coal gangue produced by coal mining, tailings from beneficiation and slag from smelting. These solid wastes are generally piled up near the mining area. They not only occupy large areas of land and induce geological disasters such as landslides and mudslides but also cause chemical pollution, spontaneous combustion, and radiation from radioactive materials due to long-term stacking. This may affect the health and safety of humans and other biological organisms. The scope of the solid waste area is determined by the ore piles, coal piles, and dumping sites (Fig. 7b).

Mine geological disasters are caused by a large number of mining wells and rock and soil deformation, as well as serious changes in the geological, hydrogeological, and natural environments of the mining area, endangering human life and property and destroying mining engineering equipment and mining resources. In this study, the geologic hazard range consists of areas with underground mining stopes and coal piles (Fig. 7c).

After the pollutants generated by the mining operation enter the soil, physical and mechanical absorption, retention, colloidal physicochemical adsorption, chemical precipitation, bioabsorption, etc. of the suspended pollutants through the soil continue to accumulate in the upper soil. When pollutants reach a certain maximum, they cause deterioration of the soil composition, cycle, properties, and functions and begin to accumulate in plants, which affects the normal growth and development of plants, decreases crop yield and quality, and ultimately affects human health. Metallic and non-metallic minerals have different effects on soil pollution. The pollution of soil by non-metallic minerals mainly occurs in coal mines and coal piles, and the buffer zone is centred on the coal mines and coal piles. Coal production activities can cause heavy metals in coal piles to enter the soil and cause pollution. Due to different types of heavy metals, the range of soil contamination is different^47^. Combining the non-metallic mineral industrial squares and coal mine-based non-metallic minerals around the heavy metal soil pollution range, the buffers are graded at 30, 200, and 1000 m (Fig. 7d1)^43^. The metal mines in Gongyi are mainly aluminium ore and iron ore. Referencing the spread range of heavy metal pollution in the soil of aluminium ore and iron ore mines^48–51^, the buffers are graded at 50, 100, 300, and 500 m (Fig. 7d2).

The four new elements in the improved evaluation system are normalized from 0 to 1 during the calculation.

#### Intelligent evaluation model

Artificial neural networks, decision trees, and SVMs were calculated using IBM SPSS Modeler software to find an intelligent model suitable for environmental assessment of the mine in the study area. Then, several models with high evaluation accuracy were selected. The SVM, CART, and C5.0 models were chosen for further comparison. The sampling points were selected randomly; 700 sampling points were selected from the area away from the mining area; 100 sampling points were selected from the mining area after random sampling by mine type, and these points were used as training samples. Non-mining evaluation scores were based on the national environmental protection standard, while the mining area scores were based on field investigation. In the field investigation, preliminary scoring of the sampling points was conducted according to mining type, mining intensity, air quality, and surrounding environment. Then, a photo of the field was taken at every sampling point in the mine, and experts were invited to further score the area according to the photo. This score is the relative score obtained by referencing the national environmental protection standard.

The index layers of the training samples were used as input, and the scores were used as the output to train the machine learning models. The trained models were applied to the entire study area, and all points except the training sample points were used for verification. After further comparison with SVM, CART, and C5.0, the evaluation accuracy rates of the three methods in the mining area and non-mining area were obtained. In the non-mining area, the model evaluation results of various land use types were compared with the national environmental protection standards. The accuracy in various land use types is shown in Table 5. In the mining area, the model evaluation score is compared with the score from the experts, and the obtained accuracy table is shown in Table 6.

**Table 5:** Accuracy of each algorithm in various land use types in non-mining areas.

Land use types	Algorithm		
	SVM (%)	CART (%)	C5.0 (%)
Mining lease	98.37	99.08	98.06
Residential land	71.57	80.15	78.45
Scenic spot	100.00	100.00	99.78
Road	99.02	97.89	97.85
Woodland	79.74	78.40	81.47
Garden	99.67	99.17	99.78
Arable land	68.63	66.36	65.73
Water	94.12	91.82	91.16
Grassland	90.85	88.05	89.22
Other land	98.04	99.08	98.49
Totals	94.80	84.55	91.90

**Table 6:** Accuracy of each algorithm in the mining area.

Algorithm	SVM (%)	CART (%)	C5.0 (%)
Accuracy	82.50	89.36	85.62

In non-mining areas, the accuracy of the SVM model is significantly better than that of the other two methods. However, in the mining area, the accuracy of the CART model is higher. Therefore, the SVM model was used to evaluate the area away from the mine, and the CART model was used to evaluate the mining area. The evaluation results of these two models were combined to obtain the evaluation map of the entire study area.

## Discussion

Comparing the former evaluation results of the first season to the evaluation results by the improved system shows that the overall difference in the new results is more obvious and that the pollution of the mining area is increased. The mountainous area in the southeast is unexplored forestland and is far away from the mining area and the city. Thus, the vegetation coverage is high, and logically, the eco-environmental quality should be the best. However, the official evaluation results do not prominently reflect this information, but the improved evaluation results of the area show good indications of an excellent environment. The difference in the evaluation results of the southwestern, northern, and central-eastern regions of the improved system is larger than that of the former systems, which is not as consistent as the results of large-area regional evaluations. Taking the northern region as an example, as a region containing different vegetation indexes, air pollution values, meteorological factors, and a large variety of land types, such as water bodies, construction land, and forestland, the results from the official evaluation methods are very similar, while the improved results can reflect the role of various factors in this region. This comparison further illustrates the accuracy and practicality of the improved evaluation system.

**Figure 8:**
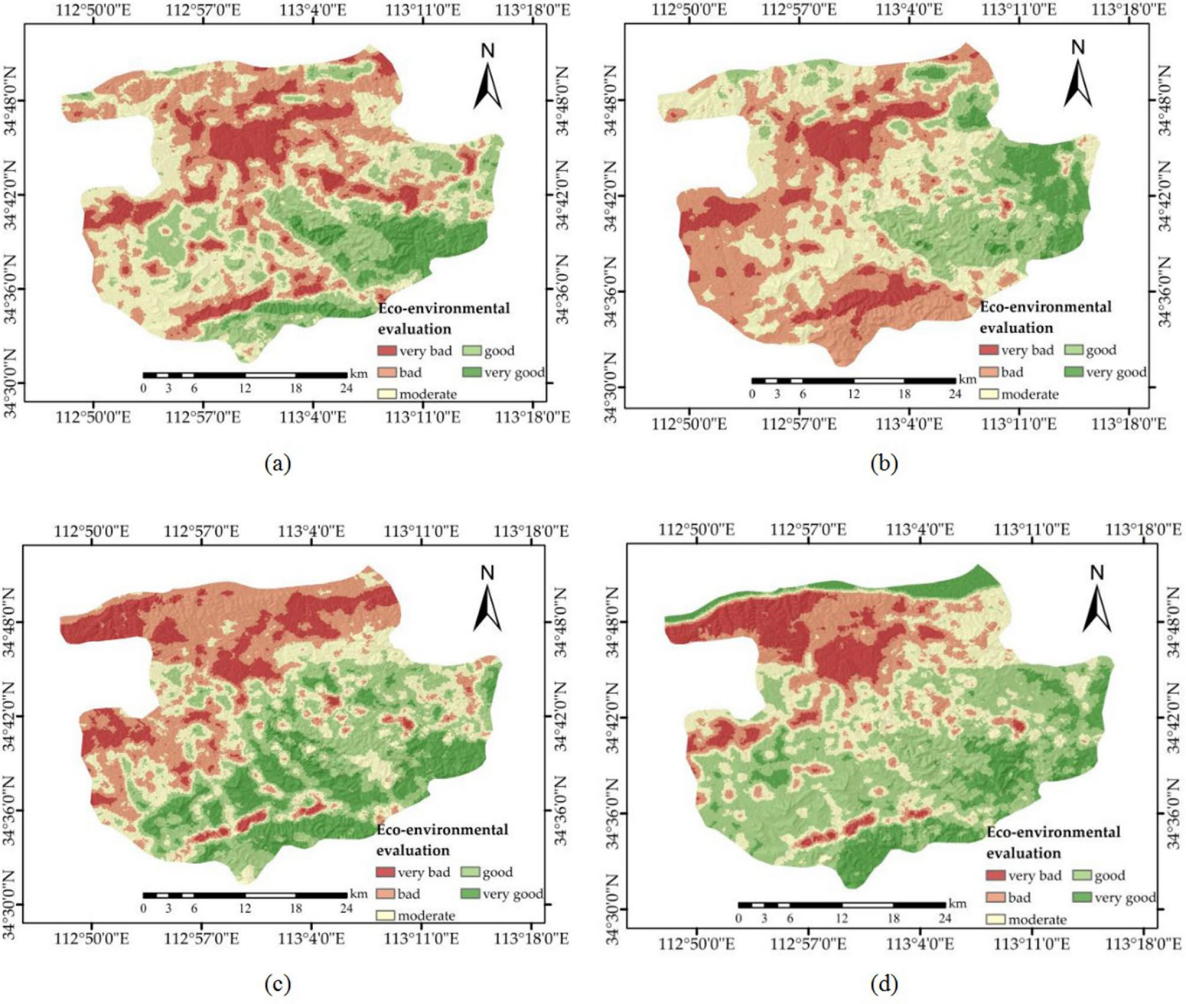
Eco-environment assessment maps of the mining area in Gongyi. (**a**) Maps of spring; (**b**) maps of summer; (**c**) maps of autumn; and (**d**) maps of winter. The Figure is created using ArcGIS ver.10.3 (https://www.esri.com/).

In addition, the meteorological data were divided into four seasons. March to May 2016 was defined as the first season (spring), June to August 2016 was defined as the second season (summer), September to November 2016 was defined as the third season (autumn), and December 2016 to February 2017 was defined as the fourth season (winter). The final composite model was used to calculate the geo-environmental assessment scores, and then, the eco-environment assessment map of the mining area in Gongyi was generated (Fig. 8). The results of the eco-environment assessment of the mining area were divided into five levels: very bad, bad, moderate, good, and very good. The following conclusions were obtained from the map: (1) the areas with very bad eco-environmental assessments are first distributed in the hilly development zone in the northwestern part of the central area, followed by the mining area in the southeastern mountainous area; (2) in addition to central residential areas and key mining areas, the moderate level of residential concentration and the small mining areas are more susceptible to climatic conditions, and such effects usually have a certain seasonality; (3) areas covered by water, such as rivers, do not necessarily have a good eco-environment; and (4) in the mountainous area of the southwest corner, except for the poor environment in the mining area, the seasonal changes in the surrounding areas are particularly obvious.

Table 7 shows the Pearson correlation coefficient values between the evaluation maps and the evaluation indexes, and the following conclusions can be obtained: (1) in spring, the quality of the eco-environment is mainly determined by the vegetation coverage index, followed by the biological abundance index, because the vegetation develops at the turn between winter and spring, so the impact of vegetation on the environment is highlighted. The relationship between this evaluation and the pollution loading index is different from the other three seasons, indicating that the changes in the AQI in the spring have less impact on the eco-environment. In this season, the area of the mining region with poor environmental quality is particularly large, which means that in the spring, the environmental quality is most affected by mining activities. (2) In summer, the impact of the vegetation coverage index and biological abundance index on the eco-environment is still the most prominent, but the impact of the vegetation coverage index in summer is much weaker than that in spring. In the mountains of the south and southwest, the environmental quality is particularly poor compared to the other three seasons, which is related to the strong negative effect of the land stress index and air pollution range. (3) In autumn, the negative effects of the land stress index and pollution loading index are more pronounced, and the negative correlation with the geologic hazard range also increases, while the river density index that should be positively correlated becomes abnormal, which leads to low environmental quality in the entire northern region in autumn. The environmental quality of the southern mountains in autumn is better than that in the other three seasons. (4) In winter, the biological abundance index has little impact on environmental quality, and the environmental quality of the surrounding areas of the key mining areas is significantly different from that inside the mining area, which means that the mining area has little impact on the surrounding environment. The river density index has significantly increased control over the environmental quality, so the environmental quality of the northern river covered area is obviously good.

**Table 7:** Pearson correlation coefficient values between the evaluation results and evaluation indexes.

Indexes	Seasons			
	Spring	Summer	Autumn	Winter
Biological abundance index	0.472	0.419	0.389	− 0.044
Vegetation coverage index	0.714	0.571	0.425	0.323
River density index	0.093	0.091	− 0.016	0.268
Land stress index	− 0.293	− 0.302	− 0.357	− 0.353
Pollution loading index	0.142	− 0.244	− 0.376	− 0.366
Air pollution range	− 0.088	− 0.108	− 0.094	− 0.110
Solid waste area	− 0.197	− 0.214	− 0.232	− 0.244
Geologic hazard range	− 0.125	− 0.133	− 0.156	− 0.150
Mine soil pollution buffer	− 0.134	− 0.112	− 0.093	− 0.110

The indicator layers in the national standard document referenced in the system are feasible for evaluating the environment of a normal area. The advantages are consistent with the integration of various factors of the classic models for evaluation, such as CIM. After considering the impact of the mine region, the combination with machine learning is more practical and objective than classic models.

## Conclusion

This paper uses national standard documents and machine learning methods to assess the ecological environment of mines. Environmental assessment maps for the entire study area and a practical environmental assessment model of mining areas were generated. The first advantage lies in the use of high spatial resolution RS images for mine interpretation and field sampling surveys to improve the interpretation results. This process is not limited to only traditional field investigations for interpretation of the mining area but also uses RS to save time and effort. Second, in terms of factor selection, this process utilizes the factors of national standards and increases the factors related to mines, adding various considerations. This method combines relevant factor layers and machine learning for environmental assessment. This strategy can be used in other regions of the world to obtain appropriate composite algorithms based on different environmental characteristics. Compared with other classic methods that use specific rules or artificially determined factor weights, such as AHP, machine learning methods are more accurate and reliable, and system flexibility and scalability are improved. However, because of the scale limitation of some specific factors, some of the factors involved in the calculation cannot be determined according to the actual situation. Nevertheless, with the addition of more comprehensive factors, a higher accuracy of the assessment model can be achieved. These problems still deserve further study. With the optimization of machine learning algorithms, algorithms based on subjective experience will reveal greater limitations, and consequently, an increasing number of machine learning algorithms will be used in the field of environmental assessment.
